# A Literature Review of Work From Home Phenomenon During COVID-19 Toward Employees’ Performance and Quality of Life in Malaysia and Indonesia

**DOI:** 10.3389/fpsyg.2022.819860

**Published:** 2022-05-19

**Authors:** Norhasni Zainal Abiddin, Irmohizam Ibrahim, Shahrul Azuwar Abdul Aziz

**Affiliations:** Department of Management, Faculty of Defence Studies and Management, Universiti Pertahanan Nasional Malaysia, Kuala Lumpur, Malaysia

**Keywords:** work from home phenomenon, COVID-19, employee performance, quality of life, anxiety

## Abstract

The purpose of this paper is to determine the performance of employees employed at home during the COVID-19 pandemic in Malaysia and Indonesia, also to examine the employee’s quality of life affected by COVID-19. The current study is aimed to critically determine the performance of employees employed at home during the COVID-19 pandemic. The author has analyzed and reviewed various sources of articles, reports, and documents from previous research and literature. The findings explain that working from home has provided advantages and disadvantages for both the employees and the organization and is responsible for the decrease in employee productivity. In addition, the findings conclude that the fact that working from home is generally not feasible because many areas of work cannot be done from home, although for many employees, working from home has provided a work-life balance.

## Introduction

Since 2019, all countries in the world are faced a global pandemic called Coronavirus Disease (COVID-19). Hence, this pandemic has become a critical challenge to human life in the world across all sectors, including health, politics, and security. It also becomes a major threat to all the organization, which has led to changes in work methods. It is more important than ever to explore the ability of companies to adapt to uncertain circumstances and deal with emerging situations related to the urgent challenges of the coronavirus outbreak and subsequent lockdowns. One of the changes in working methods implemented in responding to this pandemic by conducting Work From Home (WFH). It leads to giving tasks and responsibilities to its employees by prohibiting them from working in the office and gathering in a room. This change aims to prevent the spread of COVID-19.

**Figure 1:**
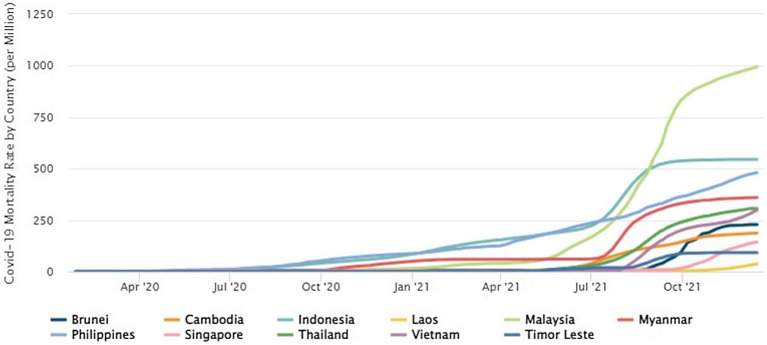
Comparison of mortality rates among Southeast Asia countries from April 2020 to October 2021 (Center for Strategic and International Studies, 2021).

From Figure 1 above, most of the countries in Southeast Asia shows a rapid surge in mortality rate around the third quarter of the year 2021. As it approaches the fourth quarter of 2021, Malaysia shows the highest mortality rate followed by Indonesia. This increasing trend has pressured both countries to take proactive measures to ensure the COVID-19 outbreak worsens.

Apart from Singapore, Southeast Asian nations have experienced some tremendous daily infections and fatalities in recent months. From 1 May and 11 July 2021, the daily average of new cases per million population in Malaysia has continued to rise exponentially. Meanwhile, Indonesia which has a population almost ten times that of Malaysia, witnessed an approximately sevenfold rise in the same metric as of 11 July 2021, compared to 1 May 2021. Furthermore, the number of daily cases has broken records in recent months, despite no major rise in the number of tests (Sasongko, 2021).

Indonesia has outnumbered India as the new COVID-19 epicentre in Asia. The country’s limited test accessibility mitigated Indonesia from demonstrating a similar jump to India throughout its worst days as a result of infection rates per million of population. Increases in the number of cases have been accompanied by increases in the number of fatalities. The increased COVID-19 cases have been followed by a dramatic spike in the mortality toll. Because of the same conditions and time, Malaysia witnessed a 24-fold rise whereas 0.13 fatalities per million population on 1st April compared to 3.13 fatalities per million population on 13th July. Despite a “mere” 6.5-fold rise over the same time, Indonesia’s death toll is currently the highest in the region. As of 13th July, Indonesia has the highest fatality rate in Southeast Asia, with 2.61 fatalities per 100 confirmed COVID-19 cases, while Malaysia shows 0.75 per cent followed by Thailand with 0.81 per cent (Sasongko, 2021).

As of June 2021, Malaysians had an 11-fold greater risk of COVID-19 infection compared to Indonesians with 21.32 cases per million in Indonesia versus 236.46 -new cases per million population in Malaysia. Nevertheless, as of July 11, this gap has only been reduced by twofold in just 1 month. This occurred despite Indonesia’s valiant attempts to expand its testing capability (Sasongko, 2021).

This research firstly intends to evaluate the effectiveness of employees working from home during the COVID-19 epidemic. Secondly, since prior studies upon the quality of life associated with health aspects have focused on several groups with particular illnesses as respondents, this research will investigate the quality of life of individuals impacted by COVID-19 who started working from home as a group of healthy participants facing psychological distress. The document and literature chosen would specifically cover the following purposes:

(1) To determine the performance of employees who were working at home during the COVID-19 pandemic in Malaysia and Indonesia.
(2) To examine the employee’s quality of life affected by COVID-19 Malaysia and Indonesia.

## Literature Review

The COVID-19 number of fatalities was again surging in various Southeast Asian nations since July 2021, as has been reported by the Center for Strategic & International Studies. The amount of new COVID-19 infections has risen as a consequence of the rapid development of the Delta variant, which might have improved its infectiousness. Lockdown restrictions have been reintroduced as a result of such. Concurrently, Indonesia has a COVID-19 fatality rate of 392 per million on August 9, making it one the worst in the southeast followed by Malaysia and the Philippines, with rates of 334 and 262 per million, respectively. Additionally, due to 4 cases of Delta variant infections among its workers, Malaysia’s parliament has put the nation under lockdown for two weeks starting 1 June 2021. Conflicts are rising throughout the public discussion, as they criticize the lockdown, claiming that this is a part of the government’s order to dodge taking responsibility for its management of the pandemic outbreak (Jeong, 2021).

**Figure 2:**
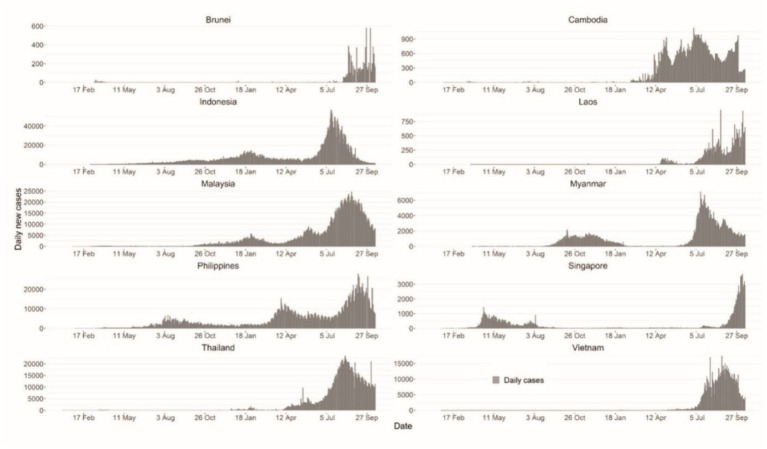
Case epidemiologic curves for all ASEAN nations from 1st January 2020 to 15th October 2021 (Rampal et al., 2021).

From Figure 2, COVID-19 infection is ubiquitous in practically all Southeast Asian nations in Galanti et al. (2021), as per current and past reports, compared to a year ago since the pandemic began. From the above graph in Figure 2, it suggests that this new wave of infection spreading very rapidly requires a larger emphasis on working remotely from home or shortly called WFH (Rampal et al., 2021).

On 25 January 2020, the very first case of COVID-19 has been identified in Malaysia. This case was originated from three Chinese citizens who had prior had direct contact with an infected individual in Singapore. On 24 January 2020, they had travelled to Malaysia through Singapore. They were then sent to Sungai Buloh Hospital in Selangor for further treatment. Following the earlier cases, larger clusters of new cases were found among individuals who had attended a huge religious (*tabligh*) mass at Masjid Sri Petaling, Selangor from 27 February to 3 March 2020. This mass had been participated by an estimated 15,000 or even more people. As of 14 April 2020, there were 4,987 confirmed cases and 82 fatalities from this cluster. The tabligh cluster was responsible for the majority of cases in Malaysia at the time. Then starting from 18 March 2020, the first total lockdown has been implemented in Malaysia to cater for this COVID-19 spread (Rampal et al., 2021).

Immediate execution, high-intensity movement restriction strategies successfully ceased the spread in response to the surge in infections. In September 2020, a sequence of infections in prisons and immigration depots, along with the easing of regulations caused by state elections, resulted in a spike in cases across the nation. Amidst continuous high-intensity suppression inside the country, the infection cases were never completely ceased, leading to a third and fourth wave of outbreaks in April and July 2021, respectively. Attributed to a combination of variables, along with more transmissible variations, ineffective management, and pandemic distress, the restriction was generally futile. Malaysia has ranked very low in terms of *per capita* fatalities and cases as compared to other ASEAN nations. Because of the severity of the infection, public health control efforts have been prioritized. Malaysia does have the second-highest testing rate, second-lowest positive test rate, and third-highest immunization rate around the region. Furthermore, recent efforts by Malaysia’s Ministry of Health to increase data transparency have resulted in Malaysia reporting the most comprehensive, publicly accessible surveillance data in the continent (Rampal et al., 2021).

On 2 March 2020, Indonesia announced their first case. Early disease management procedures in the nation included foreign travel limitations, school suspensions, movement limitations, and individual infection preventative practices that varied by area. As a result, the transmission was never completely disrupted and has always ranked among the top in the region. Nevertheless, because of the extremely high spread, a countrywide partial shutdown was implemented on 1 April 2021. The lockdown restrictions were ultimately divided into four stages, with Indonesia reaching level four as of 18 July 2021, the highest degree of lockdown in the nation. Furthermore, testing ratios have been among the lowest in the area, and test positive rates have been greater than in most other nations in the region (Rampal et al., 2021).

**Figure 3:**
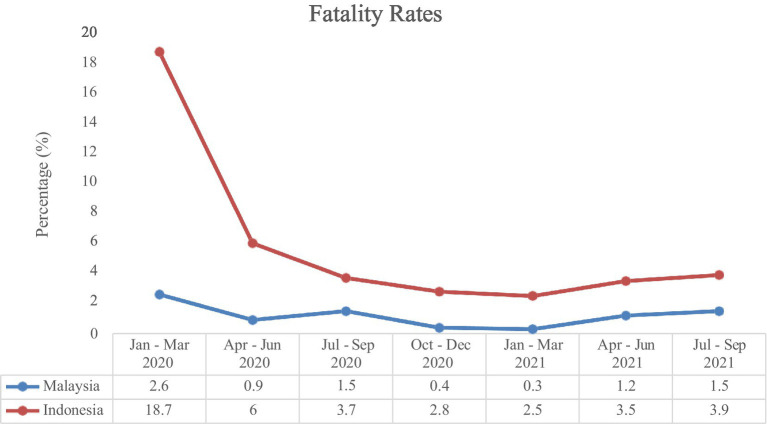
Comparison of COVID-19 fatality rates in Malaysia and Indonesia (Rampal et al., 2021). ^*^Case fatality rates, %, was delay adjusted to reflect a more valid population at risk of the case fatality.

**Figure 4:**
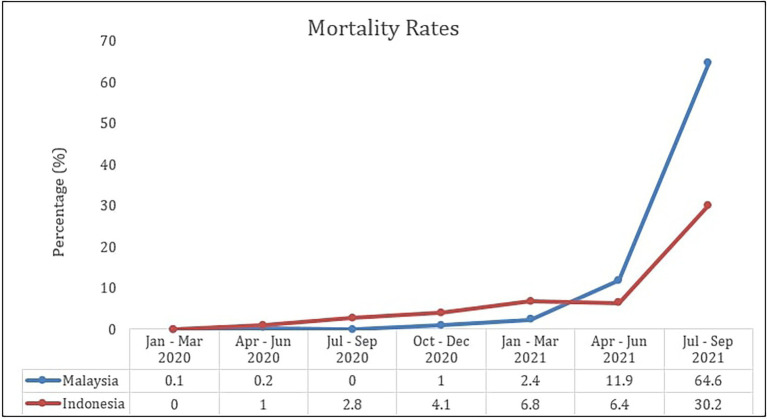
Comparison of COVID-19 mortality rates in Malaysia and Indonesia (Rampal et al., 2021). ^**^Mortality rates (per 100,000 population).

Figures 3, 4 show the comparison of fatality and mortality rates from the COVID-19 pandemic in Malaysia and Indonesia from January 2020 until September 2021. The line graph demonstrates that the fatality rate for both countries is the highest during the first quarter of 2020, and has been decreasing over the past year. However, since the first quarter of 2021, the fatality rate has started to show an increasing pattern yet again. Meanwhile, as for the mortality rate of COVID-19, a rapidly increasing pattern is shown starting from the second quarter of the year 2021. This shows that the outbreak wave has not yet subdued and more proactive preventive efforts should be taken seriously.

Employees who implement WFH are expected to be able to maintain their performance during the COVID-19 pandemic. This policy certainly has an impact on employees because the WFH situation is different from Work from Office (WFO), where WFH requires employees to be able to adapt to changes in culture or a new work environment. Work from the office and home situations are different and separate things, which work requires time and a special atmosphere to work, therefore employees need adjustment to the WFH atmosphere (Singh et al., 2020).

In Indonesia, The Ministry of Home Affairs has issued the Minister of Home Affairs Instruction regarding the community restrictions or called PPKM, which stated that certain businesses such as restaurants and shopping centers, as well as public gathering areas such as mosques, are finally allowed to operate in a limited capacity—through non-essential businesses are still obliged to implement a 100 percent work-from-home policy. Furthermore, in Malaysia, the government has decided that 80 percent of public workers, as well as 40 percent of private employees, has to work from home as one of the steps to reduce the COVID-19 infection rate.

Working from home has become prominent in many nations as a way to counter the growth of COVID-19, but it has a severe influence on mental health. The major substance was associated with both physical and mental health in persons infected with COVID-19, according to Panchal et al. (2021). Because of the lockdown, there are mental health issues in the community, which occur against a background of high rates of mental illness and substance abuse that existed previous to the current crisis. Before the pandemic, one out of every ten adults had anxiety and/or depressed symptoms, according to the report (Panchal et al., 2021). As a result, during the pandemic, roughly 4 out of 10 adults in the USA developed anxiety or depression disorder symptoms. As per a KFF Health Tracking Poll, many grown-ups have been presenting a specific harmful influence on their psychological health and well-being, like chronic insomnia (36%) or snacking (32%) due to anxiety over the outbreak, excessive alcohol intake or stimulant use (12%), and faster declining chronic health conditions (12%).

### Work From Home

Working at home is a term whereby employees may work in the comfort of their own homes. People that work from home possess better freedom in daily working time. Besides, working remotely from home offers employees to achieve a work-life balance while at the same time, supporting organizations in accomplishing tasks while reducing the danger of COVID-19 outbreak. According to Crosbie and Moore (2004), most of the salaried tasks are performed from home. Working from home offers workers more time planning flexibility, ensuring them a long-term good work-life balance. On the other side, it provides benefits for the company. Work from home is used to monitor employees’ performance so that they remain productive in completing work, in addition to giving flexible time for employees. Working at home also has advantages for businesses in terms of cost reduction such as office rent, employee litter, and other workplace support facilities that must be prepared by the company.

According to Mokhtar (2020), there are six major benefits of working from home, which are: (1) save more money and energy; (2) more family time; (3) less stress; (4) relaxed environment; (5) more productive; and (6) better internet.

Even before the pandemic outbreak, remote working has not been a frequently applied technique. While the most current survey by American Community has found that the population of US workers working from home with a minimum of 50 per cent of total working time has increased from 1.8 million in 2005 to 3.9 million in 2017, while remote working accounted for just 2.9 percent of the entire US workforce around that time. While in Europe, in 2015, just over 2 percent of people worked most of the time from home. In truth, remote working has become a privilege for the wealthier such as higher-income earners or white-collar workers (Wang et al., 2021).

Following the epidemic COVID-19, most workers had limited remotely work experience, but they and their companies were also not prepared to implement this technique. Nonetheless, the unforeseen COVID-19 outbreak in 2020 had also pressured millions of people around the world into becoming remote workers, inadvertently resulting in a global trial in telecommuting. Work from home has then rapidly become the new normal in just a few weeks (Wang et al., 2021).

The workplace setting is a crucial factor for employees to work efficiently and effectively. Employees will be more motivated to work if they work in a pleasant atmosphere, which will affect their morale and productivity. The comfort of the working space at home was among the most significant telework characteristics influencing telework outcomes. Employees’ emotions may be influenced by their work environment, which gives them security and helps them to function properly. Employees who work from home strive for a pleasant working atmosphere at home which is comparable to that of a typical workplace, such as solitude, good lighting, sufficient material. Availability of workspace, space organization, ambient conditions, as well as internet and WiFi access are mostly the important physical workplace criteria for mobile skilled employees. Another strategy for addressing work from home issues is to establish a physical setting, which involves developing a work-friendly atmosphere, such as a designated workstation with defined barriers; for example, a room with a door. This is because remote workers set physical and conceptual barriers to keep a balance between their personal and work life (Aropah and Sumertajaya, 2020).

### Employees’ Performance

The act or process of doing a task, according to the Oxford Dictionary, is known as performance. Furthermore, according to Hoffmann (1999), performance is defined as an assessed contribution to the achievement of organizational goals. Simamora (2006) agreed, stating that performance is frequently characterized as an effort that represents the effort put in. Furthermore, overall performance encompasses not only work results, but also the processes that take place while the task has been performed. The performance also refers to an organization’s goal achievement rather than an individual’s goal achievement, with the least amount of resources used to achieve the goal. The two most basic factors that define the phrase are effectiveness and efficiency. However, depending on the context in which the term is used, other aspects can be added to define the term, such as relevance, economy, efficacy, and so on.

The workplace setting is a crucial factor for employees to work efficiently and effectively. Employees will be more motivated to work if they work in a pleasant atmosphere, which will affect their morale and productivity. The comfort of the working space at home was among the most significant remote working characteristics influencing work performance. Employees’ emotions may be influenced by their work environment, which gives them security and helps them to function properly. Employees who work from home strive for a pleasant working atmosphere at home which is comparable to that of a typical workplace, such as solitude, good lighting, sufficient material. Availability of workspace, space organization, ambient conditions, as well as internet and WiFi access are mostly the important physical workplace criteria for mobile skilled employees. Another strategy for addressing work from home issues is to establish a physical setting, which involves developing a work-friendly atmosphere, such as a designated workstation with defined barriers; for example, a room with a door. This is because remote workers set physical and conceptual barriers to keep a balance between their personal and work life (Aropah and Sumertajaya, 2020).

According to the Conservation of Resources (COR) hypothesis, the resources which are physical, psychological, organizational, and emotional are important parts of well-being and fulfilment, and it is advantageous for individuals to have more resources (Hobfoll, 1989). The conservation of resources hypothesis serves as the cornerstone for this study model, which states that resources are the best collection of talents for assisting employees in completing workplace duties. Employees must create self-protective techniques to safeguard their existing resources under the current high work demand conditions faced by enterprises worldwide owing to the COVID-19 outbreak (Hobfoll et al., 2018). As per COR theory, supportive environmental, social, cognitive resources and physical resources are key assets that assist to preserve and strengthen well-being (Hobfoll, 1989; Hobfoll et al., 2018). These resources have become particularly beneficial in the present situation of the COVID-19 pandemic (Zhiqiang et al., 2021).

### Quality of Life

Pandemic outbreaks and other distressing life events might have a major profound impact on a person’s mental health and psychological wellbeing. Such mental or psychological problems that may arise include stress, anxiety, mental disorientation, social deprivation, and depression. In addition, people who are isolated because of COVID-19 infection tend to feel worried, dread, and dissatisfied. Besides, COVID-19 unpredictability has also been associated with major changes in everyday schedule, which may lead to increased stress, despair, and anxiety. Likewise, Vindegaard and Benros (2020) have written a review of the literature on the COVID-19 outbreak and personal wellbeing. At the same time, Giorgi et al. (2020) have also published a content analysis on COVID-19- linked psychological consequences throughout employment. He then concludes that COVID-19 has raised rates of sadness, sleep issues and anxiety. The previous study has examined the influence of job-related stress on a range of work practices in normal situations, however does not examine the impact of work stress on employee performance (EP) in ambiguous scenarios such as during the COVID-19 pandemic (Saleem et al., 2021).

One of the mental health difficulties linked to the work-from-home trend throughout the COVID-19 pandemic is the quality of life. The word “quality of life” pertains to objective and subjective evaluations of material, emotional, social, and physical well-being, and also personal growth as well as the levels of meaningful events, that are all influenced by a set of personal values (Karimi and Brazier, 2016).

Meanwhile, anxiety is one of a factor that is thought to have an impact on one’s quality of life. Anxiety is one of the most frequent mental health issues in people of all ages all around the world (Craske and Stein, 2016). Every year, at least 11 per cent of the world’s population suffers from anxiety as a result of a person’s inability to cope with the stress of a traumatic event (Craske and Stein, 2016). The anxiety in this study is COVID-19-related anxiety. Various groups in the global community have had anxiety symptoms as a result of the unpredictable conditions and survival resources regarded as life-threatening (Baloran, 2020; Torales et al., 2020). COVID-19-related anxiety is one of the first mental health issues to gain the attention of some researchers, who are conducting an exploratory study into assessment methods and other psychological effects or solutions induced by the pandemic (Torales et al., 2020; Anindyajati, 2021; Wang et al., 2021).

Research on work-related stress mainly focused on two major areas which explain how stress is induced. The first area that has been discussed over the years is typical job-related stress factors. The studies explored how demanding psychosocial features of work settings, such as increasing workloads, role ambiguity, lack of control, and low social support, might cause workplace stresses and degrade work performance. Meanwhile, the second area that is discussed is the environmental elements, such as evaluating how employee capabilities and their physical surroundings influence their performance and how a person-environment misfit would cause unfavorable psychological as well as physiological effects. Nevertheless, there is also another significant factor that can cause stress among the employees which are ambiguity and potentially dangerous circumstances in the workplace. As a result, components of the external surroundings conflict with employees’ capacity to perform or place unnecessary expectations on workers, hindering job performance by causing stress (Saleem et al., 2021).

## Materials and Methods

The paper will adopt a literature review of previous research articles to examine the impact of the work from home phenomenon during COVID-19 on employees’ performance and quality of life. This research uses 19 scientific articles as data sources with details in Supplementary Table S1.

In this study, the keyword of “employee performance,” “employee quality of life” as well as “work from home during COVID-19” are used. In addition, the inclusion criteria for the chosen literature are that the locations of study are Malaysia and Indonesia. Nevertheless, we exclude the literature from other countries as well as literature that are not related to working from home during this COVID-19 outbreak.

## Findings

As the COVID-19 pandemic devastates chaos in the world and alters nearly every aspect of our lives, most of us are concerned about the health of our families, friends, or ourselves. However, questions remain for people who work while being restricted to their homes due to the government’s Movement Control Order (MCO), specifically Work from Home (WFH), which applies in both Malaysia and Indonesia.

Most studies found that respondents prefer to work in an office, according to the literature examined. This is not unexpected, given most of us regard working in an office, with the correct office atmosphere, coworkers, and working hours of 8 a.m. to 5 p.m., to be natural. Working from home, on the other hand, has its own set of obstacles, with some employees finding it difficult to focus on work or work from home, particularly those with young children, elderly relatives, or other responsibilities.

Several inferences may be derived from the findings. First, as earlier research have shown, internal parameters, for example, intrinsic motivation exerts a significant influence toward shaping employee productivity (Rene and Wahyuni, 2018; Osman et al., 2021). Second, anxiety associated with COVID-19 has no significant impacts upon the quality of life and job performance of working people who perform their job from home as compared to several other aspects including technological privileges and work-life balance, which are linked to the life experiences of each person (Muliawati and Frianto, 2020; Purwanto et al., 2020). However, it is also found that one out of five individuals in Indonesia diagnosed with the COVID-19 virus would have suffered anxiety (Anindyajati, 2021).

Employees who apply WFH have characteristics which in carrying out their work has a flexible role in addition to acting as their employees as well must play the role of parent, child, and have other roles outside of their job. It has been reported that many employees are unable to balance or provide clear boundaries between personal life and work and will be vulnerable to life conflicts (Anugrah and Priyambodo, 2021). Therefore, the company has a role in making work-life balance policies for employees, where this policy can have an impact on improving employee performance or performance. According to previous research, it is critical to establish a work-life balance, such as flexible working hours, to allow employees to engage in other activities such as hobbies and personal needs outside of work. It is hoped that the implementation of a work-life balance can improve performance and employee performance (Anugrah and Priyambodo, 2021).

Work from home employees is vulnerable to family conflict, stress and frustration. When viewed from the characteristics of employees who are implementing WFH during the pandemic COVID-19 the company or organization needs to understand that employees need rewards and flexible working hours. WFH employees who have balance in life personal and work will feel satisfaction at work so that it has an impact on performance.

## Implications

The findings of this study are especially significant since they provide information on the demands of employees who have been forced to work full-time owing to the epidemic, the majority of whom had no prior WFH experience. Managers, human resources makers and workers participating in remote activities should take into account family-work conflicts, social isolation and workplace environments that distract possible obstacles as potential facilitators of WFH engagement. In times of pandemic, as COVID-19, where it contains the dissemination of the disease is fundamental, WFH is a key opportunity and can give a competitive advantage to support and improve the execution of organizations.

## Limitations

The literature review focuses on Malaysia and Indonesia; therefore, the applications to practice are limited to other countries. Therefore, future researchers can look into investigating the topic in a broader sense which should look at the perspective from other countries that might yield a different result from this study. Moreover, this study has limitations because there is no comparison between the employees who apply WFH and WFO.

## Conclusion

In Malaysia and Indonesia, the concept of working from home has been shown to have a significant impact on corporate culture and productivity. Further research into the concept of WFH and its impact on other countries will certainly focus on this discovery. The coronavirus pandemic has provided some employers with practical insight into how it affects their business and employees, even if they do not consider working from home an option for their employees. There are a variety of advantages for businesses with an increasing number of workers working from home, including flexibility, agility, enhanced employee retention, recruiting fresh talent, increased productivity, increased staff motivation, and so on. In the meantime, there are certain drawbacks to employees working from home, such as this style not suiting everyone, staff isolation, difficulty monitoring performance, home distraction, potential burnout, poor influence on mental health, and so on.

Working from home can be considered a hassle, however, this is a controversial statement. With all of the current events and technological advancements that are in line with IR 4.0, WFH has the potential to expand the scope and significance of our work platform. As a result, employees out there can benefit from working from home, such as: (1) support required; (2) training of staff; (3) technical improvement and monitoring; (4) WFH guideline; (5) direction-moving forward; and (6) appreciation.

## Originality/Value

There is a high need to understand the impact of the work from home phenomenon on employees’ performance during the pandemic. The performance of employees employed at home, which apply during a pandemic can help policymakers and various parties in getting input to deal with employees’ performance and their quality of life in respective countries. Because the COVID-19 pandemic is a world problem, it can be better than the literature on this matter to be compiled for the common good.

## Author Contributions

All authors had discussed and come out with the problem statement and research background for this study. Each author contribute substantially to this literature review paper. NA then finalize the article and came out with the conclusion. All authors listed have made a substantial, direct, and intellectual contribution to the work and approved it for publication.

## Conflict of Interest

The authors declare that the research was conducted in the absence of any commercial or financial relationships that could be construed as a potential conflict of interest.

## Publisher’s Note

All claims expressed in this article are solely those of the authors and do not necessarily represent those of their affiliated organizations, or those of the publisher, the editors and the reviewers. Any product that may be evaluated in this article, or claim that may be made by its manufacturer, is not guaranteed or endorsed by the publisher.

## Supplementary Material

The Supplementary Material for this article can be found online at: https://www.frontiersin.org/articles/10.3389/fpsyg. 2022.819860/full#supplementary-material
