# Supplementary material for: A Literature Review of Work From Home Phenomenon During COVID-19 Toward Employees’ Performance and Quality of Life in Malaysia and Indonesia
Source: Front Psychol. 2022 May 19;13:819860. doi: 10.3389/fpsyg.2022.819860 (PMC9162090; doi:10.3389/fpsyg.2022.819860)
Supplement: Supplementary file 1 [file Table_1.docx]

**Supplementary Table S1. Literature Research Results**

| **No.** | **Author, Year** | **Objectives** | **Country** | **Method** | **Sample** | **Main Findings** |
| --- | --- | --- | --- | --- | --- | --- |
| 1 | Rachmawati, et al. (2021) | To determine the extent to which Work From Home (WFH) is covered, to map workplace changes, to identify the use of ICT to support WFH, and to analyze the notion of the workplace and work systems in future cities | Indonesia | Survey: online questionnaires and focus group discussion | - Members of the Ministry of State Apparatus Empowerment of the Republic of Indonesia - Members of the Ministry of Communication and Information of the Republic of Indonesia - Members of the Regional Government of the Special Region of Yogyakarta - Consultants specializing in IT in Yogyakarta - GIS consultants in Jakarta - Google Community Mobility | - WFH is seen to have been effective enough, but it still needs lots of support from many sectors - Working from home is highly suggested for use in large cities, particularly to alleviate traffic problems and limited mobility space, as well as the necessity for office space |
| 2 | Anindyajati, G. et al. (2021) | To assess the proportion of major anxiety symptoms and their connection with COVID-19-related circumstances | Indonesia | Survey: online questionnaires | 1125 subjects in Indonesia (mostly from the western part of Indonesia) | During the COVID-19 pandemic, one out of every five Indonesians may have experienced anxiety |
| 3 | Anugrah, P., & Priyambodo, A. (2021) | To find out the role of work-life balance on the performance of employees who implement WFH during the COVID-19 pandemic | Indonesia | Literature review | 14 research articles | - Work from home employees is vulnerable to family conflict, stress and frustration. - Work-life balance can improve the performance of employees who apply WFH |
| 4 | Angin, I., & Saragih, E. (2021) | To determine the effect of work-life balance on employee performance in the presence of generations X and Y | Indonesia | Survey: online questionnaires | - 92 employee generation X - 192 employee generation | - Personal life interference work affected generation X’s work performance - Work enhancement personal life affected generation Y’s work performance |
| 5 | Foo, I., and Adam, S. (2021) | To investigate how to work from home could be used as a work-life balance intervention in the Malaysian real estate market to reduce turnover intentions | Malaysia | Survey, interview | 42 staffs in Malaysia real estate market | Work from home can help employees attain a better work-life balance and turnover intentions |
| 6 | Galanti, et al. (2021) | To examine the impact that family-work conflict, social isolation, distracting environment, job autonomy, and self-leadership have on employees' productivity, work engagement, and stress experienced when WFH during the pandemic | Indonesia | Survey: online questionnaires | 209 employees WFH full-time in Italian public and private organizations in Indonesia | - WFH productivity and engagement were negatively correlated with employee family-work conflict and social isolation, while self-leadership and autonomy were positively correlated - WFH stress was negatively associated with family-work conflict and social isolation, whereas autonomy and self-leadership had no effect |
| 7 | Nizar, et al. (2021) | To investigate the link between telecommuting work arrangements and employee performance during the COVID-19 pandemic's Movement Control Order (MCO) | Malaysia | Survey: online questionnaires | 152 respondents that Work From Home (WFH) during Movement Control Order (MCO) in Northern Malaysia | Telecommuting has a substantial impact on workplace autonomy; work-family balance and occupational stress have a significant impact on employee performance |
| 8 | Osman, et al. (2021) | To analyze direct correlations between workplace environment, peer support, employee motivation and organizational support, and employee productivity support within higher education institutions during the COVID-19 pandemic | Malaysia | Survey: online questionnaires | 579 employees in Malaysia | Employee productivity is positively influenced by the workplace environment, peer support, and employee motivation, however, employee productivity is negatively influenced by organizational support |
| 9 | Abdullah, N., et al (2020) | To determine the family value, increase in performance, isolation, disrupt the work-life balance | Malaysia | Survey: online questionnaires | 110 employees in Malaysia | Working from home has more advantages than problems, according to the majority of respondents, indicating that working from home is more convenient for employees |
| 10 | Ali, et al. (2020) | To determine the determinant for WFH facilities during MCO in Malaysia | Malaysia | Survey: online questionnaires | 363 employees in Malaysia | Six determinant elements for WFH facilities during the pandemic include work-family related, organizational support, working culture, job autonomy, the application of current technology, and the impact of social media |
| 11 | Ambikapathy, M., and Ali, A. (2020) | To analyze the influence of working from home and the difficulties that employees who work from home encounter | Malaysia | Survey: online questionnaires | 57 employees in Malaysia | Working from home has a favourable influence on cost and time savings, productivity, on-time completion of tasks, and work-life balance |
| 12 | Hashim, et al. (2020) | To assess the job satisfaction and performance of workers who work from home | Malaysia | Survey: online questionnaires | 503 administrative staff from Universiti Teknologi MARA Malaysia | Most employees were satisfied with working from home |
| 13 | Rahman, A., Jasmin, A., and Schmillen, A. (2020) | To determine the distribution of jobs most vulnerable to COVID-19 | Malaysia | Survey | Detailed data on employment patterns and the possibility to work from home and without physical proximity in Malaysia | More than half of jobs in Malaysia are particularly vulnerable to COVID-19 |
| 14 | Muliawati, T., & Frianto, A. (2020) | To look at the impact of work-life balance and job happiness on the performance of millennial employees | Indonesia | Literature review | 22 research articles | In the millennial generation, work-life balance and job satisfaction were found to be highly associated with job performance |
| 15 | Purwanto, A. et. al. (2020). | To explore the advantages and  disadvantages of working at home during pandemic COVID19. | Indonesia | Case Study: Semi-structured interview | 6 elementary school teachers in Tangerang, Indonesia | - The benefits of WFH include greater flexibility in accomplishing tasks, not needing to adhere to work hours, not having to pay for commuting or fuel costs, being able to reduce the level of stress caused by traffic jams and having more leisure time. - the drawback of WFH is that it might cause less work enthusiasm due to the need to cover electricity and internet bills, which can lead to data security issues. |
| 16 | Mustajab, D., et al (2020) | To investigate the impacts of working from home on employee productivity with a qualitative approach | Indonesia | Interview | 50 employees in Indonesia | WFH cannot be applied to all sectors that rely heavily on direct consumer services, such as health care, manufacturing, and transportation |
| 17 | Sabuhari, et al. (2020) | To investigate and assess the impact of human resource flexibility, employee competencies, organizational culture adaptation, and job satisfaction on employee performance | Indonesia | Survey: online questionnaires | 105 employees | - Employee performance was highly influenced by human resource flexibility, employee competencies, and job satisfaction - Employee performance did not improve as a result of organizational culture adaptation |
| 18 | Sulistiowati, & Komari, N. (2020) | To see how a dual-career couple's work-life balance differs when they work from home, and to see if there is a difference in WLB between male and female workers | Indonesia | Survey: online questionnaires | 100 respondents with the criteria of being a worker with a partner who also did WFH in Indonesia | -Female workers have a better work-  life balance than male workers |
| 19 | Rene, R., & Wahyuni, S. (2018) | To examine the effect of work-life balance on organizational commitment, job satisfaction, and work motivation | Indonesia | Survey | 190 employees at an insurance company in Jakarta | - Job satisfaction is significantly influenced by work-life balance - Work-life balance has no effect on dedication, organization, or motivation at work. - Individual performance is influenced by organizational commitment, job happiness, and work motivation. |

**REFERENCES**

Abdullah, N. N., Rahmat, N. H., Zawawi, F. Z., Khamsah, M. A. N., and Anuarsham, A. H. (2020). Coping with post COVID-19: can work from home be a new norm? *EJSSS* 5, 61–80. doi: 10.46827/ejsss.v5i6.933

Ali, I. M., Hamid, M. Y., Zaidi, M. A., Mat Yasin, M. F., and Hasin, M. A. (2020). Determinant for working from home facilities during the COVID-19 movement control order in Malaysia. *Eur. J. Mole. Clin. Med.* 7, 5820–5832.

Ambikapathy, M., and Ali, A. (2020). Impact and challenges towards employees working from home during COVID-19 (MCO) periode. *Int. J. Soc. Sci. Res.* 2, 97–107.

Angin, I., and Saragih, E. (2021). Pengaruh work life balance terhadap kinerja karyawan generasi X dan Y di Jakarta. *J. Emerg. Bus. Manag. Entre. Stud.* 1, 48–57.

Anindyajati, G., Wiguna, T., Murtani, B. J., Christian, H., Wigantara, N. A., Putra, A. A., ... & Diatri, H. (2021). Anxiety and its associated factors during the initial phase of the COVID-19 pandemic in Indonesia. *Frontiers in psychiatry*, *12*, 253.

Anugrah, P., and Priyambodo, A. (2021). Peran work-life balance terhadap kinerja karyawan yang menerapkan work from home (WFH) di masa pandemi COVID-19: studi literatur. Prosiding Buku Abstrak Seminar Nasional “Memperkuat Kontribusi Kesehatan Mental dalam Penyelesaian Pandemi Covid 19: Tinjauan Multidisipliner”, Fakultas Pendidikan Psikologi Universitas Negeri Malang - 3 April 2021.

Foo, I. Y. N., and Adam, S. (2021). A review on the impact of work-life balance in reducing turnover intention through work from home intervention in Malaysia real estate market. *Int. J. Acad. Res. Bus. Soc. Sci.* 11, 634–647. doi: 10.6007/IJARBSS/v11-i6/10197

Galanti, T., Guidetti, G., Mazzei, E., Zappalà, S., and Toscano, F. (2021). The impact on employee’s remote work productivity, engagement, and stress. *J. Occup. Environ. Med.* 63, e426–e432. doi: 10.1097/JOM.0000000000002236

Hashim, R., Bakar, A., Noh, I., and Mahyudin, H. A. (2020). Employee’s job satisfaction and performance through working from home during the pandemic lockdown. *AMEABRA Int. Virt. Conf. Env. Behav. Stud.* 5, 461–467.

Muliawati, T., and Frianto, A. (2020). Peran work-life balance dan kepuasan kerja terhadap kinerja karyawan milenial: studi literatur. *Jurnal Ilmu Manajemen* 8, 606–620.

Mustajab, D., Bauw, A., Rasyid, A., Irawan, A., and Akbar, M. A. (2020). Working from home penomenon as an effort to prevent COVID-19 attacks and its impacts on work productivity. *Int. J. App. Bus.* 4, 13–21.

Osman, Z., Ismail, Z., Khan, B. N. A., and Alwi, N. H. (2021). Determinants of work-from-home productivity among higher education institution employees during the COVID-19 pandemic in Malaysia. *ASEAN J. Open Dis. Learn. Spec. Issue.*, 1–13.

Purwanto, A., Asbari, M., Fahlevi, M., Mufid, A., Agistiawati, E., Cahyono, Y., et al. (2020). Impact of work from home (WFH) on Indonesian teachers performance during the Covid-19 pandemic: An exploratory study. *Int. J. Adv. Sci. Technol.* 29, 6235–6244.

Rachmawati, R., Choirunnisa, U., Pambagyo, Z. A., Syarafina, Y. A., and Ghiffari, R. A. (2021). Work from home and the use of ICT during the COVID-19 pandemic in Indonesia and its impact on cities in the future. *Sustain. For.* 13, 6760–6717. doi: 10.3390/su13126760

Rahman, A., Jasmin, A., and Schmillen, A. (2020). The Vulnerability of Jobs to COVID-19: The Case in Malaysia. Economics Working Paper.

Rene, R., and Wahyuni, S. (2018). Pengaruh work-life balance terhadap komitmen organisasi, kepuasan kerja, dan motivasi kerja terhadap kinerja individu pada karyawan perusahaan asuransi di Jakarta. *Jurnal Manajemen dan Bisnis Sriwijaya* 16, 53–63. doi: 10.29259/jmbs.v16i1.6247

Sabuhari, R., Sudiro, A., Irawanto, D., Rahayu, M., et al. (2019). The effects of human resource flexibility, employee competency. *Manage. Sc. Let.* 10, 1777–1786. doi: 10.5267/j.msl.2020.1.001

Sulistiowati, and Komari, N. (2020). Work-life balance dual career couple during COVID-19 pandemic. Proceeding Semirata International Conference.
